# Impact of regional analgesia in surgery

**DOI:** 10.1093/bjs/znab214

**Published:** 2021-06-15

**Authors:** J. Yeung, C. Small

**Affiliations:** 1 Warwick Medical School, Warwick Clinical Trials Unit, University of Warwick, Coventry, UK; 2 Hereford County Hospital, Wye Valley NHS Trust, Hereford, UK

The goal of perioperative pain management is to optimize patient experience, reduce distress, and to facilitate recovery and rehabilitation following surgery^1^. Effective pain management is fundamental to patient well-being and is an important part of the perioperative care pathway^2^. Regional analgesia (RA) describes the use of local anaesthetic and adjunct drugs to provide localized absence of nociception for surgery and postoperative recovery. Exploitation of RA is usually determined by the location and type of surgery. Techniques include neuraxial blocks, such as spinal anaesthesia and epidural anaesthesia; peripheral nerve or plane blocks, such as transverse abdominal plane (TAP), rectus sheath or paravertebral nerve blocks; and wound infiltration. The latter two can be administered as a single shot or via continuous infusion. RA can be initiated before operation, either solely or in combination with sedation or general anaesthesia, or at the end of the surgical procedure, sited by either surgeon or anaesthetist. Single-shot techniques provide analgesia for several hours after surgery, whereas continuous infusions can continue for several days as part of a multimodal analgesic regimen. Enthusiasm for RA has grown, particularly the use of peripheral nerve blocks as an alternative to neuraxial techniques. This evolution has been driven not only by the awareness of disadvantages of high-dose opiates but also increasing clinical competence and confidence in RA. Training in use of ultrasound imaging among anaesthetists is commonplace, and has advanced the precision and safety of the use of nerve blocks. Moreover, with minimal training surgeons can place peripheral nerve block catheters in the correct anatomical plane for infusion at the end of surgery. Wards and recovery areas continue to gain expertise in their ongoing management, supported by acute pain services.

The evidence that RA reduces postoperative complications and mortality is sparse, with few supporting data, except in high-risk surgical populations^3^. The biological mechanisms of benefit are multifactorial and may explain why some patients benefit more than others. Acute pain from surgical trauma activates neuroendocrine and sympathetic nervous systems, and causes a range of pathophysiological responses, including tachycardia, hypertension, hyperglycaemia, immunosuppression, decreased regional blood flow or venous stasis, and platelet aggregation^4^. RA can attenuate these pathophysiological responses to a greater extent than is seen with systemic analgesics^5^.

RA may reduce the use of opiates and other drugs with adverse risk profiles, particularly in patients who are at risk of developing significant perioperative morbidity associated with respiratory or gastrointestinal side-effects, such as the elderly and frail with hip fracture^6^. A Cochrane review^7^ concluded that, for patients undergoing abdominal aortic surgery, epidural analgesia provided better pain management, reduced myocardial infarction, time to extubation, postoperative respiratory failure, and gastrointestinal bleeding than systemic opioids. However, there was no difference in 30-day mortality.

TAP block and rectus sheath catheters have gained popularity as effective pain relief for patients undergoing intra-abdominal surgery. Their value lies particularly in their ability to provide effective relief of pain in those presenting for emergency laparotomy, and is the subject of ongoing clinical trials^8^^,^^9^. In addition, there are data to suggest that use of RA may also lead to procedure-specific benefits; for example, patency rates appear to be better in those undergoing arteriovenous fistula creation^10^.

RA attenuates the surgical stress response by blocking afferent neuronal transmission and preventing transmission to the central nervous system^11^. Persistent postsurgical pain (PPSP) affects up to 85 per cent of patients. A Cochrane review^12^ concluded that there is moderate-quality evidence that regional anaesthesia may reduce the risk of PPSP. However, these data appear to be limited to thoracotomy, caesarean sections and breast cancer surgery. High-quality clinical trials are needed to determine which is the most effective technique, the timing of intervention, and whether there are any other specific surgical populations who may benefit.

Recent preclinical evidence indicates that the systemic inflammatory response and immune disequilibrium following surgery can allow growth of metastases, highlighting the potentially important role of drugs used in the perioperative period^13^. RA may reduce the risk of cancer recurrence by minimizing the immunosuppressant effects of general anaesthetic agents and opioids. However, several large RCTs have so far failed to discern any survival benefit associated with RA^14^.

As the focus of perioperative research has moved away from merely survival after surgery to survivorship, the emphasis of perioperative analgesia has also changed from reducing pain to improving quality of recovery and promoting function. RA is not appropriate for all patients. Some find the altered sensation associated with the onset and recovery from RA more distressing than the pain avoided. Hence, a technically successful block will not always translate to enhanced patient satisfaction. RA techniques are considered to be partially or completely opiate-sparing. They serve to not only reduce unpleasant opiate related experiences, such as nausea and itching, but may also accelerate the trajectory towards the DREAMing (drinking, eating, mobilizing) paradigm^15^. Nevertheless, some RA techniques may hinder mobilization and delay ambulation^2^.

There are important methodological issues to be considered in clinical trials of RA. There is growing frustration about the heterogeneity of clinical trials of perioperative analgesic effectiveness and efficacy^16^. Most clinical trials report short-term, unidimensional, self-reported or observational scales in acute pain. Some use multidimensional tools developed for chronic pain to describe the patient experience more fully, or explore the type of pain, but most are cumbersome and lack validation in the perioperative setting. Measures designed to capture the impact of pain relief on patient experience and satisfaction have been developed, and are growing in popularity. Researchers have proposed the development of a core outcome set that is applicable, acceptable, and valuable to both patients and stakeholders^16^. However, perhaps the best way of understanding whether a particular analgesic approach confers benefits over another is to use a composite outcome measure that incorporates patient experience and functional outcome^17^.

There is a lack of robust methods to identify patients who will benefit and therefore should receive RA. The varied practice of RA highlights that decision-making around the anaesthetic and analgesic technique is often chosen driven by the preference, habit, and expertise of the anaesthetists and surgeons rather than by patient preference^18^. The resurgence of research interest in RA could help to address this apparent disconnect. In the increasingly protocolized world of enhanced recovery and perioperative management, patient characteristics and individual needs may be overlooked. Future research should consider how to personalize RA to maximize individual-patient benefit^19^. This may require a combined approach, with advanced statistical modelling incorporating patient genotypic and phenotypic data^20^^,^^21^.

*Disclosure.* JY is Director of UK Perioperative Medicine Clinical Trials Network.
